# Hepatic Deletion of Carbohydrate Response Element Binding Protein Impairs Hepatocarcinogenesis in a High-Fat Diet-Induced Mouse Model

**DOI:** 10.3390/ijms26052246

**Published:** 2025-03-03

**Authors:** Majedul Karim, Jessica Prey, Franziska Willer, Helen Leiner, Mohd Yasser, Frank Dombrowski, Silvia Ribback

**Affiliations:** Institute of Pathology, University Medicine Greifswald, Friedrich-Loeffler-Str. 23e, 17475 Greifswald, Germany; smmajedul.karim@med.uni-greifswald.de (M.K.); jessica.prey@med.uni-greifswald.de (J.P.); franzi.willer@web.de (F.W.); mohd.yasser@med.uni-greifswald.de (M.Y.); frank.dombrowski@uni-greifswald.de (F.D.)

**Keywords:** hepatocarcinogenesis, hepatocellular carcinoma, NAFLD, NASH, ChREBP, MLXIPL, PI3K/AKT/mTOR, high-fat diet, lipogenesis, liver cancer, fatty liver diseases

## Abstract

The transcription factor carbohydrate response element binding protein (ChREBP) has emerged as a crucial regulator of hepatic glucose and lipid metabolism. The increased ChREBP activity involves the pro-oncogenic PI3K/AKT/mTOR signaling pathway that induces aberrant lipogenesis, thereby promoting hepatocellular carcinomas (HCC). However, the molecular pathogenesis of ChREBP-related hepatocarcinogenesis remains unexplored in the high-fat diet (HFD)-induced mouse model. Male C57BL/6J (WT) and liver-specific (L)-ChREBP-KO mice were maintained on either a HFD or a control diet for 12, 24, and 48 weeks, starting at the age of 4 weeks. At the end of the feeding period, mice were perfused, and liver tissues were formalin-fixed, paraffin-embedded, sectioned, and stained for histological and immunohistochemical analysis. Biochemical and gene expression analysis were conducted using serum and frozen liver tissue. Mice fed with HFD showed a significant increase (*p* < 0.05) in body weight from 8 weeks onwards compared to the control. WT and L-ChREBP-KO mice also demonstrated a significant increase (*p* < 0.05) in liver-to-body weight ratio in the 48-week HFD group. HFD mice exhibited a gradual rise in hepatic lipid accumulation over time, with 24-week mice showing a 20–30% increase in fat content, which further advanced to 80–100% fat accumulation at 48 weeks. Both dietary source and the increased expression of lipogenic pathways at transcriptional and protein levels induced steatosis and steatohepatitis in the HFD group. Moreover, WT mice on a HFD exhibited markedly higher inflammation compared to the L-ChREBP-KO mice. The enhanced lipogenesis, glycolysis, persistent inflammation, and activation of the AKT/mTOR pathway collectively resulted in significant metabolic disturbances, thereby promoting HCC development and progression in WT mice. In contrast, hepatic loss of ChREBP resulted in reduced hepatocyte proliferation in the HFD group, which significantly contributed to the impaired hepatocarcinogenesis and a reduced HCC occurrence in the L-ChREBP-KO mice. Our present study implicates that prolonged HFD feeding contributes to NAFLD/NASH, which in turn progresses to HCC development in WT mice. Collectively, hepatic ChREBP deletion ameliorates hepatic inflammation and metabolic alterations, thereby impairing NASH-driven hepatocarcinogenesis.

## 1. Introduction

Hepatocellular carcinoma (HCC) poses a steadily increasing global health challenge, and is the leading cause of cancer-related death worldwide [1,2]. HCC ranks as the sixth most commonly diagnosed cancer and the third leading cause of cancer-related death [1]. HCC predominantly occurs in settings of underlying chronic liver diseases, most commonly due to hepatitis B virus (HBV) infection, hepatitis C virus (HCV) infection, alcohol abuse, or a combination of these factors [2]. The sequential progression from chronic liver disease to fibrosis and cirrhosis leads to HCC in 80% of cases, but approximately 20% of all HCC cases occur in the context of non-alcoholic steatohepatitis (NASH) [3,4,5]. It is important to note that NASH, linked to metabolic syndrome, is emerging as the leading cause of hepatocellular carcinoma (HCC), especially in Western countries [6].

Furthermore, diet is a potential lifestyle-related risk factor for HCC development [7]. Diet provides building blocks for uncontrolled tumor cell proliferation and growth. Specifically, chronic dietary fat exposure causes altered metabolic plasticity in the liver, which results in impaired glucose and lipid metabolism [8,9]. The dysregulation of de novo lipogenesis and glycolysis is at the center of many metabolic diseases such as diabetes, obesity, NAFLD, and NASH, which may further advance to hepatocarcinogenesis [10,11]. The evolution from hepatic steatosis to more severe liver diseases is accompanied by increased levels of lipotoxicity, oxidative stress, endoplasmic reticulum stress, mitochondrial dysfunction, hepatocyte injury, and cell death. These factors can trigger inflammatory responses, thereby promoting a metabolic milieu that facilitates hepatocarcinogenesis [12].

Altered cellular metabolism in cancer requires adaptation to meet the increased energy requirements during tumor initiation and progression [13]. Hence, the metabolic alteration in cancer cells undergoes elevated rates of aerobic glycolysis, de novo lipogenesis, and nucleotide biosynthesis, all of which are indispensable to sustaining their survival, growth, and proliferation [14]. Moreover, Akt/mTOR cascades stimulate de novo lipogenesis through increasing expression of lipogenic transcription factor sterol regulatory element binding protein-1c (SREBP-1c) and carbohydrate response element binding protein (ChREBP), therefore contributing to liver fat accumulation [15]. SREBP-1c and ChREBP are major transcriptional regulators that induce crucial lipogenic enzymes to facilitate lipogenesis in the liver [15]. AKT/mTOR pathway, enzymes involved in glycolysis, and de novo lipogenesis are upregulated in altered rodent hepatocytes during hepatocarcinogenesis [16,17]. In addition, emerging evidence suggests that metabolic alteration in HCC progression is positively correlated with the upregulation of transcription factor ChREBP [16,18,19,20].

ChREBP expression is most prominent in the liver, thereby contributing to the coordinated regulation of carbohydrate and lipid homeostasis in hepatocytes [21]. In addition to its role in glucose and lipid metabolism, ChREBP has been implicated in the control of other physiological processes, including inflammation, cell proliferation, and differentiation [22]. Furthermore, hepatic ChREBP deletion protects mice from carbohydrate-induced fatty liver, but high-fat diet (HFD) leads to increased hepatic fat accumulation within 12 weeks in hepatic ChREBP-deficient mice [23]. However, the molecular pathogenesis of ChREBP-related hepatocarcinogenesis in response to prolonged HFD feeding in the mouse model remains to be explored. Therefore, our present study investigated the contribution of ChREBP to NASH-related HCC tumorigenesis in the HFD-induced mouse model.

## 2. Results

### 2.1. HFD Induced Body and Liver Weight Gain in WT and L-ChREBP-KO Mice

**Figure 1:**
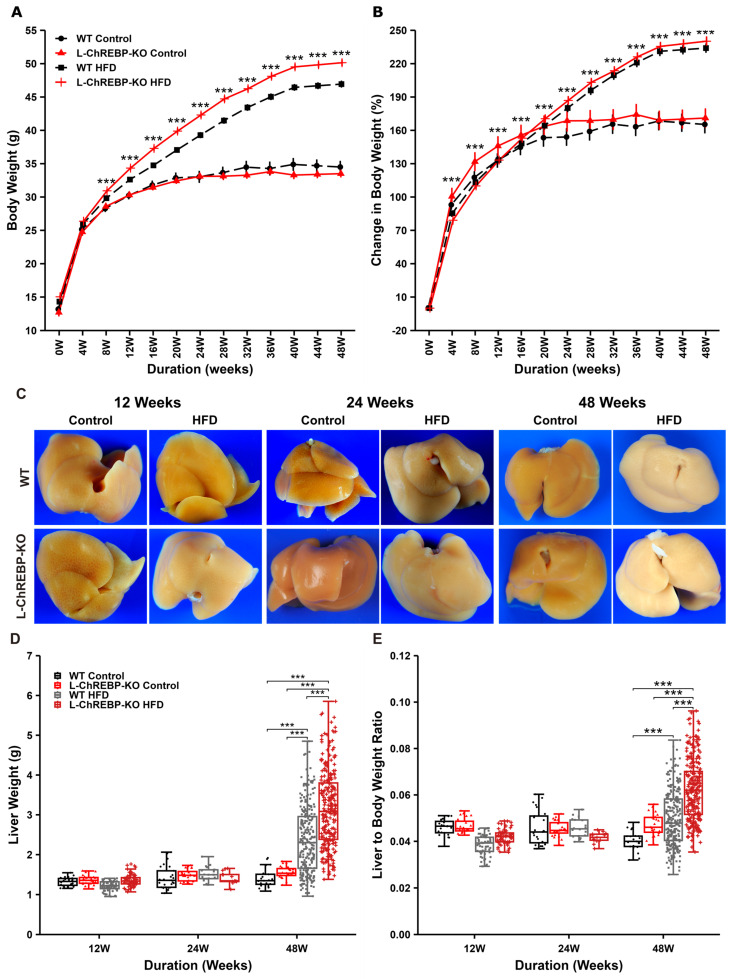
HFD feeding resulted in diet-induced obesity and hepatomegaly in WT and L-ChREBP-KO mice over a 48-week period. (**A**) Body weight changes of mice over 48 weeks, starting at 4 weeks of age. (**B**) Percent change in body weight from baseline. (**C**) Gross morphology of liver in each group. (**D**) Changes in liver weight in each group over 48 weeks. (**E**) Liver-to-body weight ratio in each group ((**A**,**B**,**D**,**E**): *n* = 264 per group in HFD; *n* = 25 per group in control). Values of the data are expressed as mean ± SEM. Significant differences are indicated as follows: *** *p* < 0.001 vs. the control group.

To determine the role of prolonged HFD feeding, mice were maintained on either HFD or a control diet for 12, 24, and 48 weeks. Mice fed an HFD displayed a steady increase in body weight from 8 weeks onwards (Figure 1A and Figure S2), while there was no notable change in body weight after 16 weeks in a 48-week period with the control diet (Figure 1A). As illustrated in Figure 1B, there were significant effects of HFD feeding on percent change in body weight, with an average increase in body weight of 164.635 ± 5.005%. Gross livers from mice maintained on HFD for 12, 24, and 48 weeks showed progressive changes in pale brownish coloration, with minimal changes in the size observed at 12 and 24 weeks (Figure 1C). The HFD feeding induced a significant increase in liver weight at 48 weeks, as well as a significant amount of lipid accumulation compared with controls (Figure 1D), as manifested by the appearance of a pale brownish color (Figure 1C).

Moreover, the liver-to-body weight ratio was not significantly different in 12- and 24-week mice, but prolonged exposure to HFD feeding induced hepatomegaly, as evidenced by an increased liver-to-body weight ratio in both WT and L-ChREBP-KO mice (Figure 1E). This was due to a significant increase in liver weight with respect to total body weight. However, short-term HFD exposure for 12 weeks and 24 weeks significantly increased the body weight of both WT and L-ChREBP-KO mice (Supplementary Figure S2). This gradual increase in body weight significantly played a role in the development of obesity and NAFLD in the HFD group over a 48-week period.

### 2.2. Increased Blood Glucose, Triglyceride, Total Cholesterol, Glycogen, ALT, and AST Levels in WT and L-ChREBP-KO Mice on an HFD

**Figure 2:**
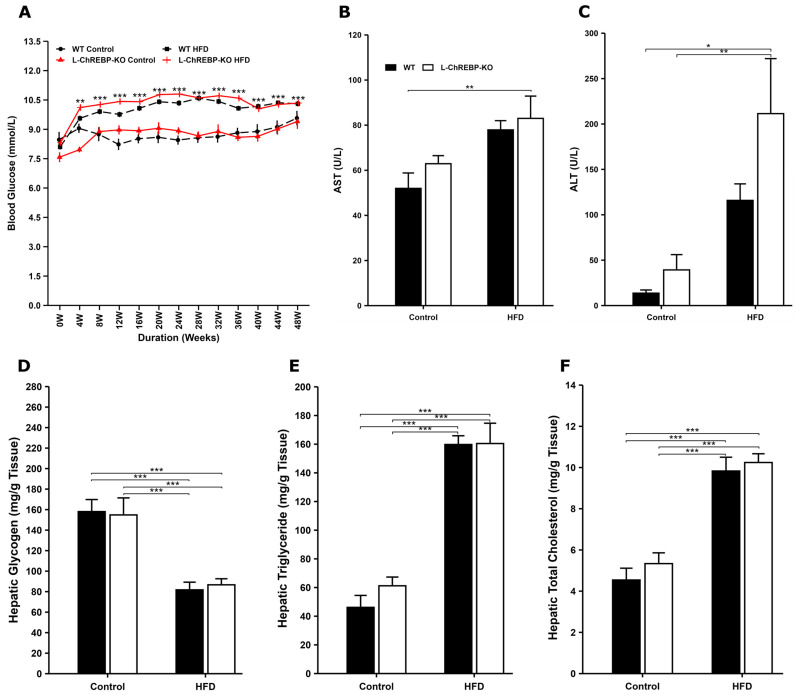
Effect of diet and genotype on biochemical parameters. (**A**) Changes in blood glucose levels over a 48-week period among the groups (*n* = 264 per group in HFD; *n* = 25 per group in control). (**B**,**C**) Serum ALT and AST levels in WT and L-ChREBP-KO mice at the age of 48 weeks (*n* = 5–6 per group). AST: Aspartate Aminotransferase; ALT: Alanine Aminotransferase. (**D**–**F**) Hepatic glycogen, triglyceride, and total cholesterol levels in 48-week WT and L-ChREBP-KO mice (*n* = 4–6 per group). Values of the data are expressed as mean ± SEM. Significant differences are indicated as follows: * *p* < 0.05, ** *p* < 0.01, and *** *p* < 0.001 vs. the control group.

The liver plays a crucial role in blood glucose homeostasis within a relatively narrow range. We measured basal blood glucose levels at approximately 7.6 mmol/L at the start of the study, with no statistical differences between the groups. In mice fed with HFD, basal blood glucose levels significantly increased from 4 weeks onward among the groups, whereas no change was observed in both WT and L-ChREBP-KO mice maintained on the control diet (Figure 2A and Figure S3). After the 48-week period, basal blood glucose levels reached around 10.3 mmol/L in mice kept on HFD, but control mice showed around 9.5 mmol/L at 48 weeks. These findings suggest a progressive deterioration in blood glucose metabolism in the HFD groups.

Increased dietary fat intake has been considered an unhealthy dietary component, which has a harmful effect on the liver. To determine the liver function, we performed alanine aminotransferase (ALT) and aspartate aminotransferase (AST) assays. The hepatocellular injury was not observed in both WT and L-ChREBP-KO mice within a 24-week period, as evidenced by the normal range of serum ALT and AST levels (Supplementary Figure S4). We observed that there was a substantial increase in the serum ALT and AST levels in liver-specific ChREBP-KO mice compared to WT control mice at 48 weeks (Figure 2B,C). These results indicate that long-term HFD feeding aggravated the liver function, thus contributing to NAFLD development.

The liver plays a crucial role in maintaining blood glucose levels via glycogenesis and glycogenolysis. Moreover, glycogen accumulation in hepatocytes was evident in WT and L-ChREBP-KO mice, as indicated by the PAS staining. There was a gradual accumulation of hepatic glycogen within a 24-week period, although statistical significance was absent between the control and HFD groups (Supplementary Figure S4). Interestingly, our study confirms that quantitative analysis of liver tissue displayed higher glycogen storage in L-ChREBP-KO and WT mice maintained on a control diet than the HFD groups (Figure 2D). We also observed occasional nuclear glycogen accumulation in both WT and L-ChREBP-KO mice at 24 and 48 weeks.

Hepatic triglyceride accumulation in NAFLD is associated with an increased risk of obesity and dyslipidemia, which are the hepatic manifestations of the metabolic syndrome [24]. The fatty acids in hepatic triglycerides are derived from dietary sources and de novo lipogenesis. A gradual increase in hepatic triglyceride accumulation was observed over the 24-week period (Supplementary Figure S4); however, no statistical significance was noted during this timeframe. As expected, we observed that mice fed with long-term HFD exhibited significantly higher hepatic triglyceride storage, whereas the control diet did not induce triglyceride storage in both WT and L-ChREBP KO mice, resulting in a highly significant difference between the control and HFD groups (*p* < 0.05) in Figure 2E. Therefore, the progressive triglyceride accumulation led to NAFLD in mice maintained on an HFD.

The liver is involved in the coordination of lipid metabolism and thus plays a significant role in the development of dyslipidemia. The primary source of hepatic total cholesterol could be synthesized cholesterol in the liver, while the rest comes from dietary intake. Our results demonstrate that mice fed a long-term HFD exhibited significantly higher hepatic total cholesterol levels, while the control diet did not cause an increase in total cholesterol levels in both WT and L-ChREBP KO mice (Figure 2F). As a result, the sustained accumulation of total cholesterol over a 48-week period contributed to the development of NAFLD in mice kept on an HFD. There was no difference in the hepatic total cholesterol levels within a 24-week period between the control and HFD groups (Supplementary Figure S4). Taken together, chronic dyslipidemia and hepatic lipid overload caused an escalating vicious circle, resulting in detrimental consequences on liver metabolism and function, and ultimately promoting liver damage in WT mice.

### 2.3. Prolonged Dietary Fat and Increased Lipogenesis Resulted in Hepatic Fat Accumulation

**Figure 3:**
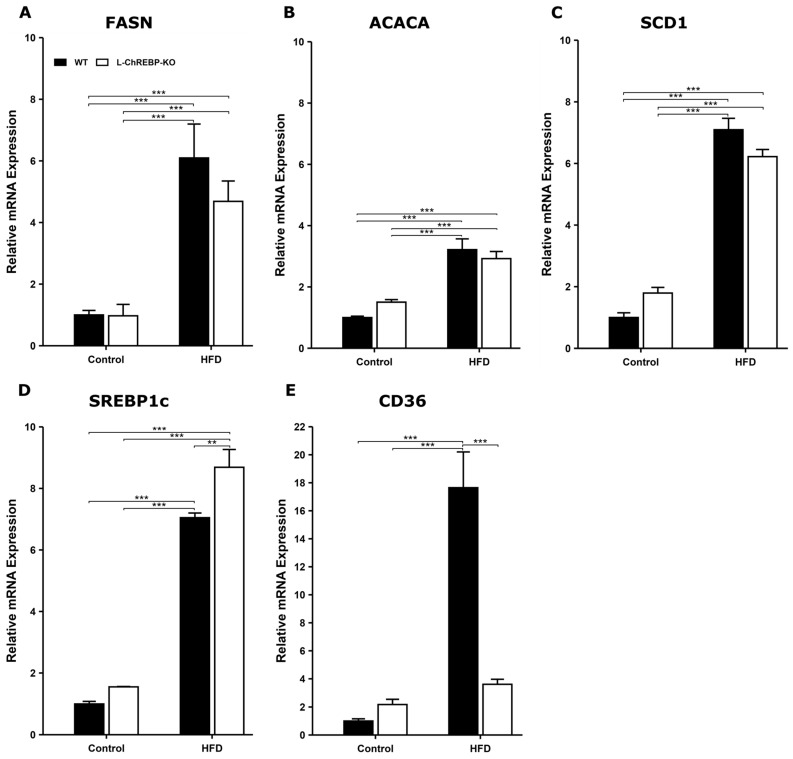
Effects of HFD on the expression of hepatic lipid metabolism-related genes in 48-week mice. (**A**–**C**) Expression of key genes (FASN, ACACA, and SCD1) involved in de novo. (**D**) Lipogenic transcription factor SREBP-1c expression in 48-week mice. (**E**) Intracellular fat transport-associated gene CD36 expression in response to HFD feeding (*n* = 3–4 per group). Values of the data are expressed as mean ± SEM. Significant differences are indicated as follows: ** *p* < 0.01, and *** *p* < 0.001 vs. the control group.

To gain further insight into the underlying causes of hepatic steatosis induced by chronic HFD consumption, we investigated the expression profiles of major regulatory genes necessary for hepatic lipid metabolism (FASN, ACC1, SCD1, SREBP-1c, and CD36). Previous studies found that hepatic de novo lipogenesis is regulated by diet composition at the transcriptional level [25]. Our study confirms that 12- and 24-week HFD-fed mice showed significantly higher expression of lipogenic genes involved in de novo lipogenesis compared to the control mice (Supplementary Figures S5 and S6). Thus, the progressive increment in the expression of lipogenic enzymes played a key role in the hepatic fat accumulation in the 48-week period (Figure 3). The mRNA expression level of hepatic SREBP-1c in liver-specific ChREBP deficient mice was progressive; 12-week mice showed an increment of SREBP-1c expression (Supplementary Figure S5) and subsequently, SREBP-1c expression further revealed significantly higher activity in hepatic ChREBP-deficient mice at 48 weeks (Figure 3D). As shown in Figure 3E, WT mice increased fat uptake due to chronic dietary fat intake over the 48-week period, resulting in hepatic fat accumulation (Figure 3E). The fat uptake gene CD36 expression was not significantly different among the 12-week and 24-week groups (Supplementary Figures S5 and S6). The results suggest that the ChREBP-dependent pathway is involved in hepatic fat transportation in WT mice. Taken together, prolonged HFD feeding led to hepatic fat accumulation through both ChREBP-dependent and independent lipogenic pathways in WT and L-ChREBP-KO mice.

### 2.4. Long-Term HFD Feeding Induced Inflammation and Altered Metabolic Pathways in NASH Progression

**Figure 4:**
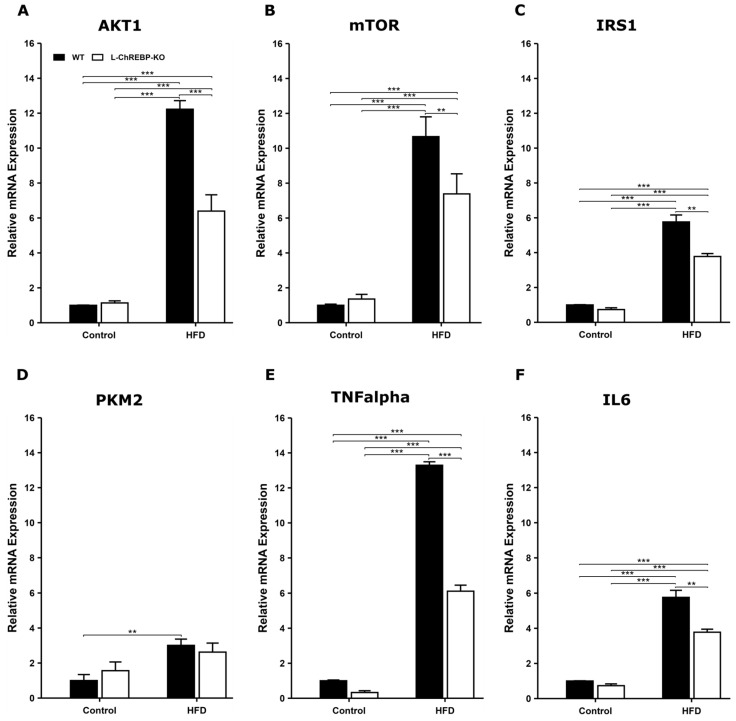
Changes in expression levels of AKT1/mTOR, insulin signaling, glycolysis, and inflammation-associated genes in 48-week mice. (**A**,**B**) The mRNA expression of AKT1 and mTOR in 48-week mice. (**C**) Insulin signaling gene expression levels. (**D**) Altered expression of PKM2; (**E**,**F**) Upregulation of inflammation-related genes, TNFalpha and IL6 (*n* = 3–4 per group). Values of the data are expressed as mean ± SEM. Significant differences are indicated as follows: ** *p* < 0.01, and *** *p* < 0.001 vs. the control group.

Prolonged HFD feeding can increase the hepatic fat accumulation, thus contributing to the NAFLD development. In mice fed with HFD, the expression levels of AKT and mTOR genes in WT mice were significantly higher than in L-ChREBP-KO mice over 12-week and 24-week periods (Supplementary Figures S5 and S6). Subsequently, AKT and mTOR expression levels were significantly higher in mice maintained on the HFD over the 48-week period, therefore AKT/mTOR pathway contributed to the progressive NAFLD development (Figure 4A,B). In agreement with previous studies [26], the mRNA expression level of insulin signaling candidate IRS1 was increased in HFD groups in 12-week and 24-week periods (Supplementary Figure S5). In contrast, we did not observe significant differences in IRS1 mRNA levels among the WT and L-ChREBP-KO mice at 48 weeks, which were maintained on HFD compared to the control groups (Figure 4C). The mRNA expression levels of PKM2 showed unaltered gene expression in 12-week and 24-week mice (Supplementary Figure S6). However, a significant expression was evident at 48 weeks, suggesting HFD-induced alterations in the glycolytic pathway (Figure 4D).

Furthermore, we investigated the progression of NAFLD to the more aggressive non-alcoholic steatohepatitis or NASH development in both animals exposed to HFD feeding. The mRNA expression of IL6 and TNFalpha was significantly higher in 48-week mice in response to prolonged high-fat feeding (Figure 4E,F). However, there was no change in the inflammatory markers over the 24-week period between WT and liver-specific ChREBP-deficient mice (Supplementary Figure S6).

### 2.5. Prolonged HFD Feeding Led to Steatosis and Steatohepatitis

**Figure 5:**
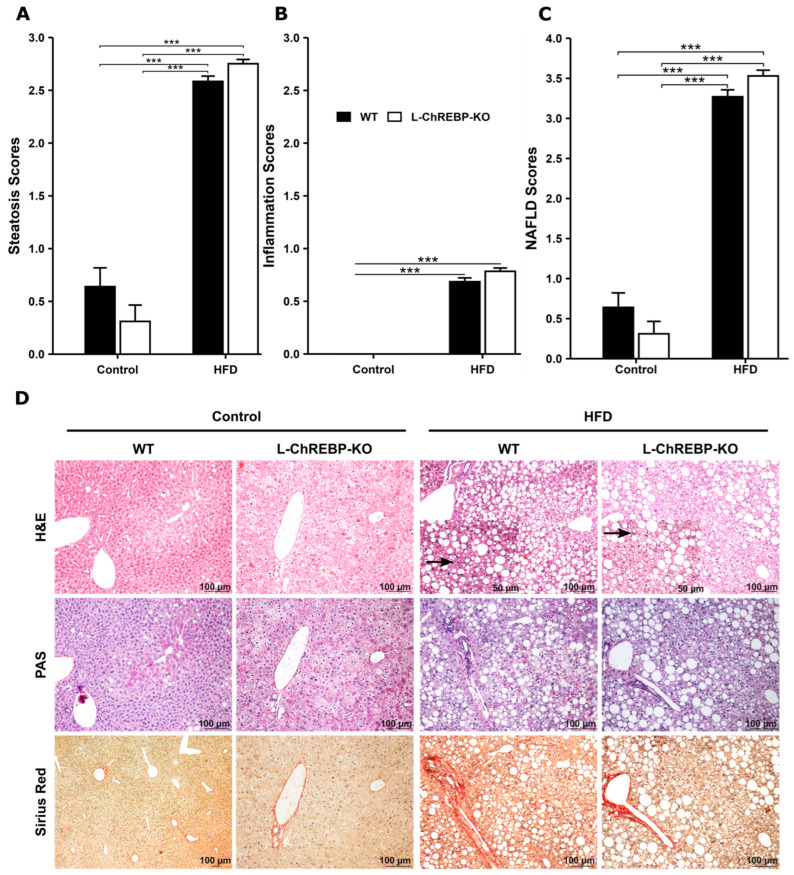
Histological assessment of NAFLD/NASH at 48 weeks in WT and L-ChREBP-KO mice. (**A**) Histological scoring of HFD-induced steatosis at 48 weeks within each group (*n* = 264 per group in HFD; *n* = 25 per group in control). (**B**) HFD feeding led to inflammation in mice at 48 weeks within each group (*n* = 264 per group in HFD; *n* = 25 per group in control). (**C**) NAFLD scores at 48 weeks within each group of mice (*n* = 264 per group in HFD; *n* = 25 per group in control). (**D**) Representative images of H&E-stained histological slides illustrating the enhanced fat accumulation in mice in response to HFD exposure. The black arrow in WT and L-ChREBP-KO indicates steatohepatitis in the HFD group. Scale bar: 100 µm. Values of the data are expressed as mean ± SEM. Significant differences are indicated as follows: *** *p* < 0.001 vs. the control group.

To explore the pathological progression of NAFLD and NASH, we investigated hepatic fat accumulation exposed to HFD feeding. Histological analysis in the liver sections of HFD mice demonstrated a significant increase in lipid droplets and inflammation compared to mice maintained on a control diet (Figure 5D). We exhibited lipid-containing hepatocytes, microsteatotic hepatocytes as shown by the small lipid droplets, and macrovesicular steatosis as indicated by the large vacuoles or droplets. Mice maintained on HFD revealed significantly higher lipid accumulation at 48 weeks, but steatosis was absent in mice fed on a control diet over 48 weeks (Figure 5A). A progressive fat accumulation in hepatocytes was observed in both WT and L-ChREBP-KO mice, with 12-week-old mice showing 5–10% fat accumulation, 24-week-old mice exhibiting 30–40% fat storage, and 48-week mice showing up to 80–100% fat accumulation (Figure 5D). Inflammation was only observed in HFD mice at 48 weeks, while L-ChREBP-KO mice on a control diet of the same age remained free of inflammation (Figure 5B). Prolonged HFD feeding induced NAFLD, resulting in differences in NAFLD scores between control and HFD mice at 48 weeks (Figure 5C). Fibrosis was absent in both WT and hepatic ChREBP-deficient mice at 48 weeks, indicating long-term HFD feeding facilitated NAFLD/NASH development without manifestation of hepatic fibrosis (Figure 5D).

### 2.6. Molecular and Metabolic Changes in NAFLD Progression

**Figure 6:**
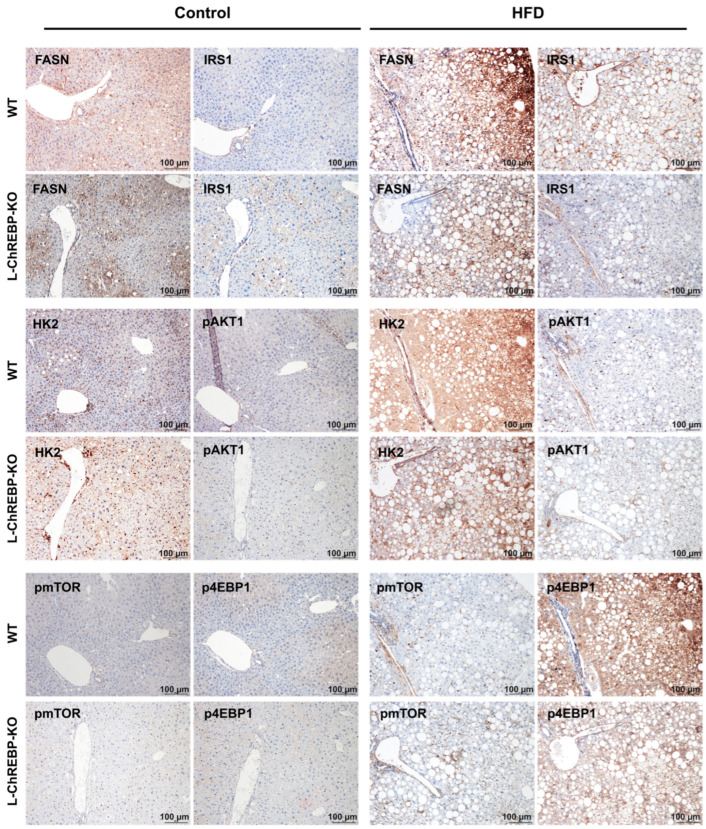
Representative images of immunohistochemical staining in liver tissue of WT and L-ChREBP-KO mice at 48 weeks. Activation of AKT/mTOR pathway in WT. Alteration in the expression of HK2, PKM2, and IRS1 in WT mice. Upregulation of de novo lipogenesis candidates FASN expression in WT mice. Scale bar: 100 µm.

To explore the effects of hepatic ChREBP loss on the progressive development of NAFLD in HFD-induced mice, the expression of AKT/mTOR, glycolysis, de novo lipogenesis, and insulin signaling pathway candidates was investigated with immunohistochemical staining. The upregulated expression of the AKT pathway played a key role as a downstream effector of the insulin signal cascade to induce NAFLD development (Figure 6). In addition, the alteration in the expression of HK2, PKM2, and IRS-1 was observed at 48 weeks in WT mice (Figure 6). The increased expression of de novo lipogenesis candidate FASN significantly contributed to hepatic fat accumulation in WT and L-ChREBP-KO mice (Figure 6). Moreover, we observe that the progressive alterations in the metabolic pathways, such as AKT/mTOR, insulin signaling, glycolysis, and de novo lipogenesis, involved in the NAFLD development at 12 and 24 weeks (Supplementary Figures S7 and S8). Collectively, the results suggest that the upregulated activity of the AKT pathway, de novo lipogenesis, and glycolysis altogether resulted in metabolic disturbances, which facilitated the development of NAFLD/NASH-driven HCC in WT mice.

### 2.7. Cellular Proliferation, Distinct Morphological, Molecular, and Metabolic Alteration of HCA and HCC in WT and L-ChREBP-KO Mice

**Figure 7:**
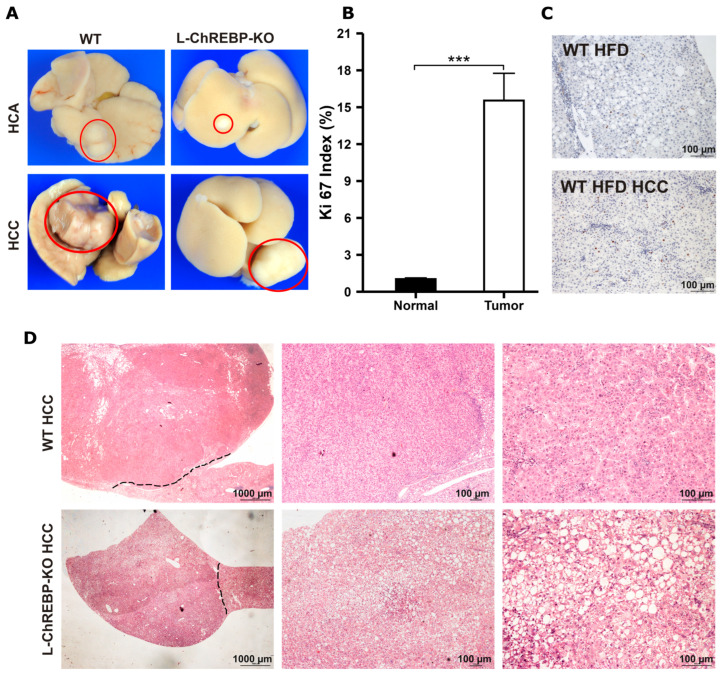
Tumor development, hepatocyte proliferation activity, and histological staining of WT and L-ChREBP-KO mice. (**A**) Representative macroscopic appearance of the liver with HCA and HCC in WT and L-ChREBP-KO mice. The red circle marks the HCA and HCC development within liver. (**B**) Ki-67 proliferation index in tumor tissue of WT mice (*n* = 55 in WT normal liver; *n* = 4 in WT tumor). The proliferation index for the L-ChREBP-KO HFD group was excluded from statistical analysis because only one tumor was observed in this group. (**C**) Representative immunohistochemical image with Ki-67 staining in WT HFD and WT HFD tumor tissue. The proliferation index of Ki-67-stained hepatocytes was determined at 40× magnification and the nuclei were counted in three randomly chosen fields, which was approximately 2000 cells per section. Positive hepatocytes for Ki-67 are stained in brown. (**D**) H&E staining of WT and L-ChREBP-KO mice. The black dashed line demarcates HCC development from normal liver (left picture). Magnifications for H&E staining: 10×, 40×, and 100×. Scale bar: 1000 µm for 10× and 100 µm for 40× and 100×. Values of the data are expressed as mean ± SEM. Significant differences are indicated as follows: *** *p* < 0.001 vs. the control group.

To investigate the role of ChREBP in hepatocyte proliferation, we counted the Ki-67-positive cells in WT and L-ChREBP-KO mice. The Ki-67 proliferation index was significantly higher in steatotic livers compared to mice kept on a control diet in a 48-week period (*p* < 0.05; Supplementary Table S4). As expected, no statistical difference was found in the hepatic Ki-67 index between the mice at 12 weeks and those at 24 weeks (Supplementary Table S4). The Ki-67 index of hepatocellular adenomas (HCA) was observed in both WT and L-ChREBP-KO mice maintained on HFD (WT: 6.69 ± 0.22%; L-ChREBP-KO: 6.33 ± 0.80%). As shown in Figure 7B, a high Ki-67 proliferation index was observed in WT tumor tissue compared to normal liver (Normal: 1.02 ± 0.09% vs Tumor: 14.75 ± 0.17%).

HCCs were observed as mass-forming tumors at the surface of the liver with a diameter of 8.58 ± 1.99 mm in WT and 5.42 mm in L-ChREBP-KO mice (Figure 7A). Besides, HCA appeared with its tumor nodules with a diameter of 2.23 ± 0.18 mm in WT and 2.24 ± 0.22 mm in L-ChREBP-KO (Figure 7A). HCCs were clearly visible with basophilic hepatocytes that were enlarged and arranged in solid trabecular patterns, with trabeculae thicker than two cell layers. These carcinomas showed enlarged nuclei, prominent nucleoli, mitotic activity, and sometimes areas of necrosis, reflecting significant changes characteristic of hepatocellular malignancy (Figure 7D). In addition, the frequency of HCA in WT and L-ChREBP-KO mice was 4.17% and 2.65%, respectively, but there was no statistically significant difference between the groups. Consistent with previous studies where the incidence of HCC development in HFD mice was only 2.5% [27], our study revealed an HCC frequency of around 1.5% in WT and 0.37% in L-ChREBP-KO HFD mice (Supplementary Table S5). WT mice fed with a control diet showed spontaneous HCC development, but not in L-ChREBP-KO mice, further providing evidence about the role of ChREBP in the pathogenesis of HCC development. The mRNA expression of ACACA and SCD1 was not different between the tumor and non-tumor tissue (Supplementary Figure S9). We observed that the mRNA levels of de novo lipogenesis, AKT/mTOR, and inflammation showed significantly higher expression in WT tumors compared to the adjacent normal liver tissue (Supplementary Figure S9).

### 2.8. Activation of AKT/mTOR, De Novo Lipogenesis, and Glycolysis Pathways in Mice HCC

**Figure 8:**
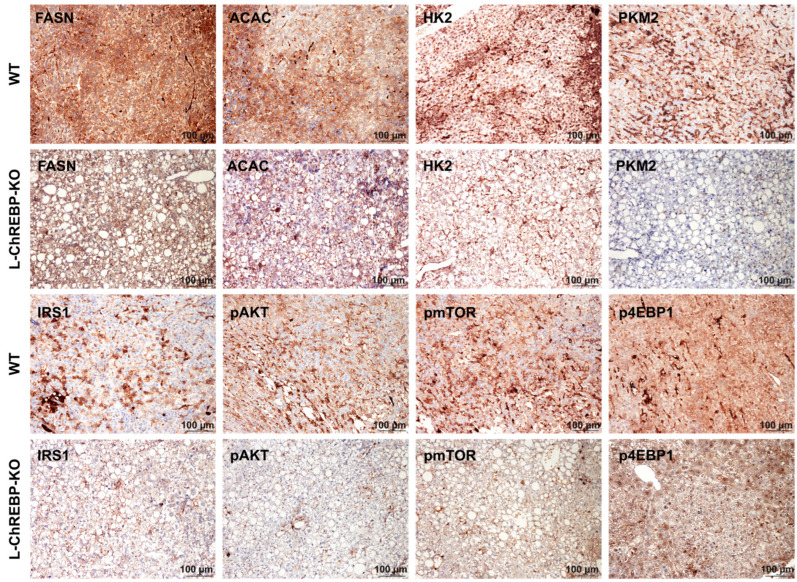
Representative immunohistochemical staining in HCC tissue of WT and L-ChREBP-KO mice. Alteration in the expression of HK2, PKM2 and IRS1 in WT. Upregulated expression of pAKT, pmTOR, and p4EBP1 in WT. Increased expression of de novo lipogenesis proteins, ACAC, and FASN, in WT mice. Scale bar: 100 µm.

The altered lipid metabolism can reprogram both cancer cells and the adjacent non-tumor cells [28]. Immunohistochemical expression of p-Akt, p-mTOR, and p-4EBP1 proteins was examined to determine the pathological role of ChREBP in NASH-related HCC development. The expression of p-Akt, p-mTOR, and p-4EBP1 was significantly higher in WT HCC compared to L-ChREBP-KO mice, suggesting a link between ChREBP activity and the AKT/mTOR pathway (Figure 8). As expected, the expression of de novo lipogenesis candidate protein FASN and ACAC was upregulated to meet the energy demand in altered hepatocytes of WT HCC (Figure 8). In addition, the increased glycolysis provided energy to the proliferative hepatocytes in HCC, as indicated by the higher HK2 and PKM2 expression in WT (Figure 8). The expression of insulin signaling candidate IRS1 showed higher expression in WT compared to hepatic ChREBP-deficient mice. These results provide evidence that the activation of ChREBP-associated metabolic pathways was involved in the regulation of hepatocyte proliferative activity and therefore contributed to hepatocarcinogenesis in WT mice.

## 3. Discussion

Cancer cells metabolize lipids to facilitate crucial oncogenic functions, including tumor initiation, progression, and growth. Evolving evidence suggests that dietary fat plays a crucial role in HCC pathogenesis [7]. The transcription factor ChREBP is one of the key factors in enhancing lipid synthesis in the liver [21]. Aberrant lipid metabolism, along with the upregulation of ChREBP, plays an essential role in both the development and progression of HCC [11,29]. However, the molecular mechanisms driving ChREBP activation and its association with HCC development are limited and inconclusive. Specifically, understanding the molecular pathophysiology of ChREBP involvement in HCC development in response to prolonged HFD feeding could pave the way for advancements in novel treatments against this deadly liver cancer. A diet-induced animal model has the potential to mimic the human condition, which can recapitulate the physiological, metabolic, and histological alterations in mice [30]. In this study, we fed mice for a period of 12, 24, and 48 weeks with either a control diet or a 45% (total kcals) fat diet to induce hepatic steatosis and/or inflammation in WT and liver-specific ChREBP knockout mice.

Chronic consumption of calorie-dense HFD is associated with obesity, therefore leading to the development of metabolic dysfunction and NAFLD in mice [31]. Consistent with previous studies [32,33], our study demonstrates that chronic HFD consumption increased morphometric parameters (body weight, liver mass, and fat pads). Another study reported that liver-ChREBP-KO and WT mice were maintained on an HFD for 12 weeks, during which there was no significant change in total body mass and lean mass between the two groups [23]. On the contrary, our 48-week study shows that hepatic ChREBP deletion in HFD-fed mice led to significant body and liver weight gain compared to both WT HFD and control (WT and L-ChREBP-KO) mice. These results indicate that prolonged HFD feeding exacerbated the NAFLD within the 48-week period after liver-specific ChREBP knockout. Despite the fact that liver-specific ChREBP deletion resulted in increases in both body and liver weight when fed an HFD, the absence of ChREBP in the liver may still confer a protective effect against the initiation and progression of HCC.

Furthermore, chronic HFD feeding led to the development of hepatic steatosis in mice, which was accompanied by increased blood glucose levels. Interestingly, we demonstrate that blood glucose levels were significantly higher in hepatic ChREBP-KO HFD mice compared to both WT HFD and control mice at 24- and 48-week, indicating that HFD feeding in hepatic ChREBP loss contributed to an increase in the blood glucose levels. As a result, hepatic ChREBP deletion impaired the regulation of systemic blood glucose levels in mice. Liu et al. showed in a study in mice that HFD feeding can augment glycolysis, as evidenced by the elevated mRNA levels of rate-limiting enzymes governing glycolysis, thereby accelerating NAFLD progression [34]. Another study investigated the effect of both a high carbohydrate diet (HCD) and HFD on hepatic ChREBP-deficient mice [23]. Their results reveal that hepatic ChREBP deletion prevents hepatic steatosis in HCD mice, whereas HFD feeding exhibits hepatic fat accumulation in liver-specific ChREBP-deficient mice [23]. Similarly, our study shows that HFD feeding resulted in hepatic fat accumulation at the age of 12 weeks in both WT and L-ChREBP-KO mice. However, the mRNA levels of SREBP-1c were notably elevated in hepatic ChREBP knockout mice compared to WT mice, indicating that SREBP-1c expression was likely a compensatory response to the absence of ChREBP for hepatic steatosis and contributed in the NAFLD development in our study. In addition, the elevated mRNA level of de novo lipogenesis candidates (FASN, ACACA, SCD1) significantly contributed to the hepatic fat accumulation in both animals exposed to prolonged HFD feeding. Moreover, HCC almost exclusively develops in the context of chronic liver inflammation, which can be caused by prolonged alcohol intake, viral hepatitis, or an unhealthy diet [35]. Our results provide evidence that the NAFLD status further exacerbated with inflammation to develop NASH in WT mice, as evidenced by the significantly higher expression of TNFalpha and IL-6 in WT mice compared to the L-ChREBP-KO group. Interestingly, our results provide evidence that the low inflammatory recruitment in hepatic ChREBP-deficient mice substantially contributed to impaired HCC development despite having HFD-induced steatosis in hepatic ChREBP-KO mice.

Moreover, the liver-specific ChREBP-KO exhibits a 23% reduction of fatty acid oxidation in liver, where carbohydrates were predominantly utilized as substrate, further supporting our study’s finding that mice fed with HFD developed NAFLD [23]. This study provides evidence substantiating the role of high dietary fat intake in contributing to the onset of fatty liver disease and associated metabolic alterations in HFD mice. The dietary fat uptake significantly contributed to ectopic hepatic fat accumulation in 48 weeks WT mice, as observed by the higher expression of CD36 gene expression. The elevated hepatic fatty acid transporter CD36 expression can result in dyslipidemia, thereby contributing to the NAFLD-related HCC progression [36,37]. Consequently, both dietary source and de novo lipogenesis exerted a significant role in promoting NAFLD-driven HCC development in WT mice.

However, aberrant hepatocyte proliferation in chronic liver disease is a significant contributing factor to the development of HCC. A recently published study reported that hepatic ChREBP overexpression in mice exhibits liver hypertrophy that is associated with increased hepatocyte proliferation rate in ChREBP-transduced hepatocytes [18]. The increased ChREBP activity contributes to the initiation and progression of hepatocarcinogenesis, as evidenced by a higher number of BrdU-positive hepatocytes and enhanced expression of cell cycle proteins [18]. Our study also exhibited that there was a progression of Ki-67-positive hepatocytes from 12 weeks to 48 weeks, where HFD feeding promoted increased hepatocyte proliferation. WT mice fed with HFD showed a higher hepatocyte proliferation rate compared to control mice (WT and L-ChREBP-KO) in 48 weeks, suggesting prolonged HFD feeding promoted initiation and progression of HCC development. Besides, the expression of ChREBP-regulated genes, which are involved in lipogenesis, increased within the tumor, further supporting the enhanced ChREBP activity and involvement during HCC development [18]. Our data demonstrate that glycolytic HK2 and PKM2 expression were upregulated in cancer cells which emphasized the diet-associated alterations in hepatic metabolism in the context of HCC. Furthermore, another study reported that HFD alters AKT-mTOR signaling pathways, resulting in an increase in phosphorylated mTOR expression in mice liver tissues [38]. Our study reveal that hepatic ChREBP deletion displayed reduced activity of AKT/mTOR cascades in response to prolonged HFD-feeding. Considering the downregulation of AKT/mTOR in L-ChREBP-KO mice, we provide the evidence to support the role of hepatic ChREBP deletion to impair HCC development.

## 4. Materials and Methods

### 4.1. Experimental Animals and Diets

All animal studies were carried out in accordance with the Animal Policy and Welfare Committee of the University Medicine Greifswald, Germany. Male C57BL/6J wild type (ChREBPWT or ChREBP+/+) and liver-specific ChREBP KO knockout (L-ChREBP KO) mice with deletion in exon 1 were used in our experiments [23]. Liver-specific ChREBP KO mice were generated by breeding floxed mice (ChREBP fl/fl, originally generated by Prof. Dr. Chan, Baylor College of Medicine, Houston) with mice expressing Cre recombinase in the liver (Alb-Cre mice: B6.Cg-Speer6-ps1Tg(Alb-cre)21Mgn/J, a generous gift from Prof. Dr. rer. Nat. Gerald Willimsky, Berlin School of Integrative Oncology, Berlin). The resultant homozygous progeny was liver-specific ChREBP knockout, without showing expression of ChREBP in the liver (Supplementary Figure S1). ChREBP WT and L-ChREBP KO were confirmed through PCR genotyping and western blotting (Supplementary Figure S1). Animals were provided with ad libitum access to food (ssniff Spezialdiäten GmbH, Soest, Germany) and tap water, unless otherwise specified. Mice were housed in a controlled room at 20–24 °C temperature with 55–65% humidity under the 12-h light-dark cycle.

Experimental animals were divided into three groups (3 months, *n* = 178; 6 months, *n* = 84; and 12 months, *n* = 578) for two different diets: control or high-fat diet (HFD) (Table 1). The experimental mice were weaned and randomly assigned to either HFD or a control diet starting at the age of 4 weeks. Dietary obesity was induced in HFD mice by allowing free access to a high-fat diet (45 kJ% fat, 20 kJ% protein, 35 kJ% carbohydrates, choline content 920 mg/kg). Control groups were fed a control diet (10 kJ% fat, 20 kJ% protein, 70 kJ% carbohydrates, choline content 920 mg/kg). Detailed composition of mouse diets is shown in the Supplementary Table S3.

### 4.2. Weight and Blood Sugar Measurements

The mice body weight and blood sugar levels were recorded at the onset of maintaining on control or HFD. The blood sugar level and body weight were subsequently monitored on a monthly basis until the day of sacrifice. Blood samples were taken from the tip of the tail veins, and were measured with a handheld glucometer (ACCU-CHEK, Roche Diagnostics, Indianapolis, IN, USA).

### 4.3. Tissue Collection

The animal sacrifice was carried out under anesthesia (400 mg/kg body weight ketamine and 40 mg/kg body weight xylazine) at the end of the feeding period. The blood was collected from the abdominal aorta for further biochemical assays. The liver samples (middle lobe) were snap-frozen using isopentane (2-methylbutane) cooled with liquid nitrogen and then stored at −80 °C for further experiments. The remaining liver was immediately collected in 4.5% formaldehyde for histological and immunohistochemical analysis. The liver tumor tissue was collected separately and stored at −80 °C for further analysis.

### 4.4. Histology

The livers were preserved in 4.5% formalin (*w*/*v*) and subsequently embedded in paraffin. The paraffin-embedded blocks were then serially sectioned at 6 µm, and stained with Hematoxylin and Eosin (H&E), Sirius Red, and Periodic acid Schiff (PAS) reaction and examined by a light microscope for histological analysis. NAFLD scoring was performed according to a general NAFLD scoring system for rodent models [39]. The severity of steatosis was graded based on the percentage of the total area affected, with the following scores: 0 for <5%, 1 for 5–33%, 2 for 34–66%, and 3 for >66%. For inflammation scoring, a focus (not a row) was defined as a cluster of ≥5 inflammatory cells per field. Five distinct fields were evaluated at 100× magnification and categorized as follows: 0 for <0.5, 1 for 0.5–1.0, 2 for 1.0–2.0, and 3 for >2.

### 4.5. Immunohistochemistry

Formalin-fixed and paraffin-embedded liver tissues were serially sectioned with 1–2 µm thickness and were stained for hexokinase II, pyruvate kinase M2 (PKM2), phosphorylated AKT (pAKT), phosphorylated mammalian target of rapamycin (mTOR), acetyl-CoA carboxylase (ACAC), fatty acid synthase (FASN), and Ki-67. All primary antibodies with detailed information are listed in Supplementary Table S2. The immunohistochemical reactions were assessed semi-quantitatively by comparing the intensity in tumor with corresponding surrounding normal liver tissue. Negative controls were stained without any primary antibody.

### 4.6. Liver Morphology and Hepatocyte Proliferation Analysis

HCA and HCC were identified in the liver macroscopically as space occupying lesions. HCAs were detected when the lesions were sharply demarcated from the surrounding liver parenchyma. HCCs were arranged in a trabecular pattern and were characterized by lesions with trabeculae thicker than three cell layers in at least two separate areas, the presence of high numbers of mitotic figures, and a diameter of larger than 3 mm. Hepatocyte proliferation was expressed based on the Ki-67 index (number of positive hepatocytes/total number of hepatocytes × 100%).

### 4.7. Biochemical Assays

Blood samples were collected from WT and L-ChREBO-KO mice and centrifuged at 12,000 RPM for 15 min to obtain serum samples. Specimen serums were stored at −80 °C until analysis. The activities of serum enzyme alanine aminotransferase (ALT) and aspartate aminotransferase (AST) levels were determined using the commercial assay kits (Cat. No. MAK052 for ALT; Cat. No. MAK055 for AST, Sigma Aldrich, St. Louis, MO, USA). The ALT and AST assays were performed as per the manufacturer’s instructions. The metabolic parameters, including glycogen, triglyceride, and total cholesterol were measured using commercial assay kits (Cat. No. ab169558 for glycogen, Abcam, Cambridge, UK; Cat. No. E-BC-K261-M for Triglyceride; and Cat. No. E-BC-K109-M for Total Cholesterol, Elabscience, Houston, TX, USA). The WT and L-ChREBP-KO mice liver tissue homogenate were prepared and the levels of glycogen, triglyceride, and total cholesterol were determined according to the manufacturer’s instructions.

### 4.8. RNA Isolation, cDNA Synthesis, and Quantitative RT PCR

RNA was extracted from liver tissue lysate using an RNA extraction kit (Cat. No. PP-210S, Jena Bioscience GmbH, Jena, Germany) and then was reverse-transcribed with random primers using a cDNA synthesis kit (Cat. No. K1642, Thermo Fisher Scientific, Waltham, MA, USA). The gene expression analysis was performed with Syber-based real-time qPCR (qRT-PCR) in a StepOnePlus™ Real-Time PCR System (Thermo Fisher Scientific, USA). The qRT-PCR was performed in triplicate, and the mRNA expression levels were normalized to 18S rRNA. The sequences of the primers used are listed in Supplementary Table S1. Relative differences in the expression of the candidate genes in control and knockout animals were determined using the 2^−ΔΔ^Ct method.

### 4.9. Western Blot (WB) Analysis

For western blot analysis, the frozen whole liver tissue lysate was used to perform lysis using RIPA lysis buffer (0.5% Nonidet P-40, 0.1% sodium deoxycholate, 150 mM NaCl, and 50 mM Tris-HCl at pH 7.5). After homogenization, lysates were centrifuged at 15,000 RPM for 15 min and the supernatant (i.e., total protein) was taken. The protein concentration was measured using a BCA protein assay kit (Cat. No 23227, Pierce, Thermo Fisher, USA). The lysates were resolved by SDS-PAGE, followed by transferring to a nitrocellulose membrane and blocking in a blocking buffer (5% non-fat dry milk in TBST). The blocking buffer was used to dilute the primary antibody and the membrane was then incubated with the respective primary antibody at 4 °C overnight with gentle agitation. Removal of the unbound primary antibody was carried out by washing the membranes thrice with TBST for 10 min each at room temperature. The membrane was subsequently incubated for 1 h at room temperature with a secondary antibody. The membrane was further washed with TBST and was proceeded to visualize the signals using the LICOR Odyssey^®^ scanner. 

### 4.10. Statistics Analysis

All statistical analysis and graphics were performed using R 4.3.0 [40] with the RStudio Integrated Development Environment [41] with ggplot2 [42], tidyverse [43], rstatix [44], dplyr [45], car [46], and xlsx [47] packages. All experimental data were expressed as the mean ± standard error of the mean (SEM) unless otherwise stated in the figure legend. The data were plotted to assess the normality distribution. A non-parametric test was performed when data distribution was not normal, followed by a post hoc test. Data analysis was performed by two-way analysis of variance (ANOVA) or analysis of covariance (ANCOVA) with normally distributed data. When statistical significance was detected, a post hoc Tukey HSD test was performed to adjust for multiple comparisons. Liver weight was analyzed via ANCOVA with body weight as a covariate. Student’s *t*-test was used to compare two groups of data. Fisher’s exact test was performed to assess the association between the genotype and/or diet for the HCC development. *p*-values of < 0.05 were considered to indicate statistical significance for all comparisons, and *p*-values > 0.05 were not reported.

## 5. Conclusions

In summary, our findings provide evidence that the altered AKT/mTOR signaling cascades, increased glycolysis, enhanced de novo lipogenesis and fat uptake, and heightened inflammation, all together contributed to the development of NASH-related HCC in WT mice. These metabolic and proliferative alterations were significantly impaired in liver-specific ChREBP deletion. Therefore, ChREBP functions as an oncogene in the initiation and progression of HCC development in response to chronic exposure to HFD feeding in WT mice. Despite having obesity with HFD feeding, hepatic ChREBP deletion impairs hepatocarcinogenesis and thus seems to have a protective role against HCC development.

## Figures and Tables

**Table 1:** Experimental and control groups.

	HFD			Control		
	12-Week	24-Week	48-Week	12-Week	24-Week	48-Week
Wild Type C57BL6	*n* = 64	*n* = 17	*n* = 264	*n* = 25	*n* = 25	*n* = 25
L-ChREBP KO	*n* = 64	*n* = 17	*n* = 264	*n* = 25	*n* = 25	*n* = 25

## Data Availability

All results generated or analyzed during this study are included in this published article and Supplementary Materials. Data and materials will be made available upon request via email to the corresponding author.

## Supplementary Materials

The following supporting information can be downloaded at: https://www.mdpi.com/article/10.3390/ijms26052246/s1.
