# Supplementary material for: Hepatic Deletion of Carbohydrate Response Element Binding Protein Impairs Hepatocarcinogenesis in a High-Fat Diet-Induced Mouse Model
Source: Int J Mol Sci. 2025 Mar 3;26(5):2246. doi: 10.3390/ijms26052246 (PMC11900174; doi:10.3390/ijms26052246)
Supplement: Supplementary file 1 [file ijms-26-02246-s001.zip › ijms-3462960-supplementary.pdf]

Hepatic deletion of carbohydrate response element binding protein impairs hepatocarcinogenesis in a high-fat diet-induced mouse model

Stutus of long-term HFD feeding in mouse model and validation of hepatic ChREBP deletion

Most of the mice fed a HFD appeared healthy until the end of the feeding period in 48 weeks groups. Unfortunately, three mice from WT group (n=264) died unexpectedly at 16, 24 and 40 weeks. Additionaly, five mice from the WT group required perfusion at different time points before the end of feeding period due to biting injuries or other health issues. In the L-ChREBP-KO 48-week group (n=264), two mice suddenly died at 20 and 28 weeks. Since we housed four mice per cage and as a result, four other mice from L-ChREBP-KO 48-week group sustained biting injuries, which were also perfused before the end of the feeding period.

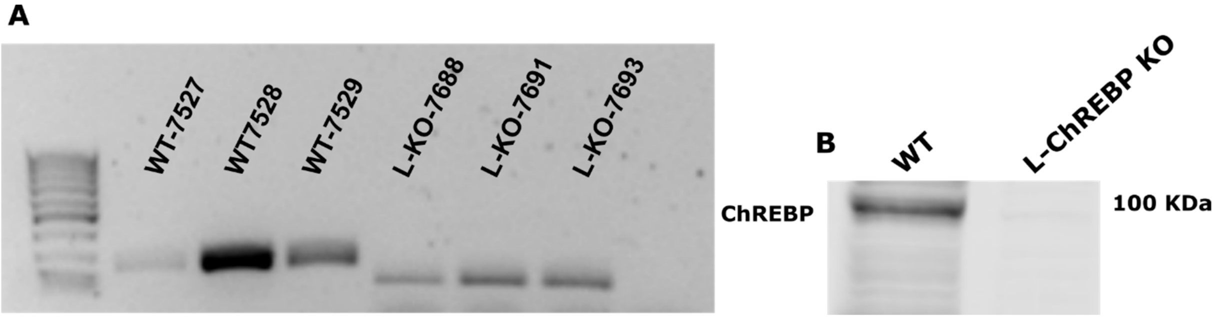

Supplementary Figure S1: (A) PCR genotying using tail genomic DNA to identify hepatic ChREBP knockout mice. PCR products of WT (Lane 1 -3) and hepactic ChREBP knockout (Lane 4-6) mice. (B) Confirmation of liver-specific ChREBP knockout through Western blot using liver tissue lysates. WT: Wild type; L-ChREBP KO: Liver specific ChREBP knockout.

Supplementary Table S1: qPCR Primer List

Gene Name	Forward	Reverse
AKT1	ATGAACGACGTAGCCATTGTG	TTGTAGCCAATAAAGGTGCCAT
mTOR	ACCGGCACACATTTGAAGAAG	CTCGTTGAGGATCAGCAAGG
FASN	CTGCCACAACTCTGAGGACA	CGGATCACCTTCTTGAGAGC
ACACA	ATGGGCGGAATGGTCTCTTTC	TGGGGACCTTGTCTTCATCAT
SCD1	AAAGCCGAGAAGCTGGTGAT	TACAAAAGTCTCGCCCCAGC
SREBP1c	GATGTGCGAACTGGACACAG	CATAGGGGGCGTCAAACAG
CD36	ATGGGCTGTGATCGGAACTG	GTCTTCCCAATAAGCATGTCTCC
IL6	GATGTGCGAACTGGACACAG	CATAGGGGGCGTCAAACAG
TNFα	ACTTCGGGGTGATCGGTCCCC	GGTTTGCTACGACGTGGGCTAC
IRS1	CGATGGCTTCTCAGACGTG	CAGCCCGCTTGTTGATGTTG
PKM2	GCCGCCTGGACATTGACTC	CCATGAGAGAAATTCAGCCGAG
18s RNA	AGTCCCTGCCCTTTGTACACA	CGATCCGAGGGCCTCACTA

Immunohistochemistry

Formalin-fixed and paraffin-embedded serial liver sections in 1-2 μm thickness were manually stained for aldolase, hexokinase II, pyruvate kinase M2 (PKM2), phosphorylated/activated AKT (pAKT), mammalian target of rapamycin (mTOR), phosphorylated/activated ribosomal protein S6 (pRPS6), acetyl-CoA carboxylase (ACAC) and BrdU. For antigen retrieval, a citrate buffer of pH 6.0 was used. Endogenous peroxidase was cleared with 1 % hydrogen peroxide, and positive reactivity of primary antibodies was performed by the HRP polymer and DAB as the chromogen substrate (Dako, Glostrup, Denmark).

The immunohistochemical reactions were assessed semi-quantitatively by comparing intensity in CCF or tumour with corresponding surrounding unaltered liver tissue. Negative controls were stained without any primary antibody.

Supplementary Table S2: The antibody list for the immunohistochemistry:

Protein	Host and clonality	Dilution	Cat. No.	Company
pAKT	Rabbit mAB	1:100	4060	Cell Signaling Technology
pmTOR	Rabbit mAB	1:100	2976	Cell Signaling Technology
p4E-BP1	Rabbit mAB	1:800	2855	Cell Signaling Technology
pERK1/2	Rabbit mAB	1:100	4370	Cell Signaling Technology
IRS-1	Mouse mAB	1:100	Sc-8038	Sant Cruz
HK2	Rabbit mAB	1:4000	22029-1-AP	Proteintech
PKM2	Rabbit mAB	1:400	4053	Cell Signaling Technology
FASN	Rabbit polyclonal	1:1000	10624-2-AP	Proteintech
ACAC	Rabbit mAB	1:400	3676	Cell Signaling Technology
ChREBP	Rabbit polyAB	1:500	NB400-135	Novus Biologicals

Supplementary Table S3: Composition of Control and High Fat Diet

**Crude Nutrients**	**Control Diet**	**High FAT Diet (HFD)**
Crude Protein	18.2 %	20.8
Crude Fat	4.1 %	23.8
Crude Fiber	4.8 %	5.5

Crude Ash	5.4 %	4.4
Starch	25 %	8.2
Sugar	32.8 %	21.5
**Additives**	**per kg**	**per kg**
Vitamin A [IE/IU]	15,000	17,500
Vitamin D3 [IE/IU]	1,500	1,700
Vitamin E [mg]	150	175
Vitamin C [mg]	30	35
Copper [mg]	12	14

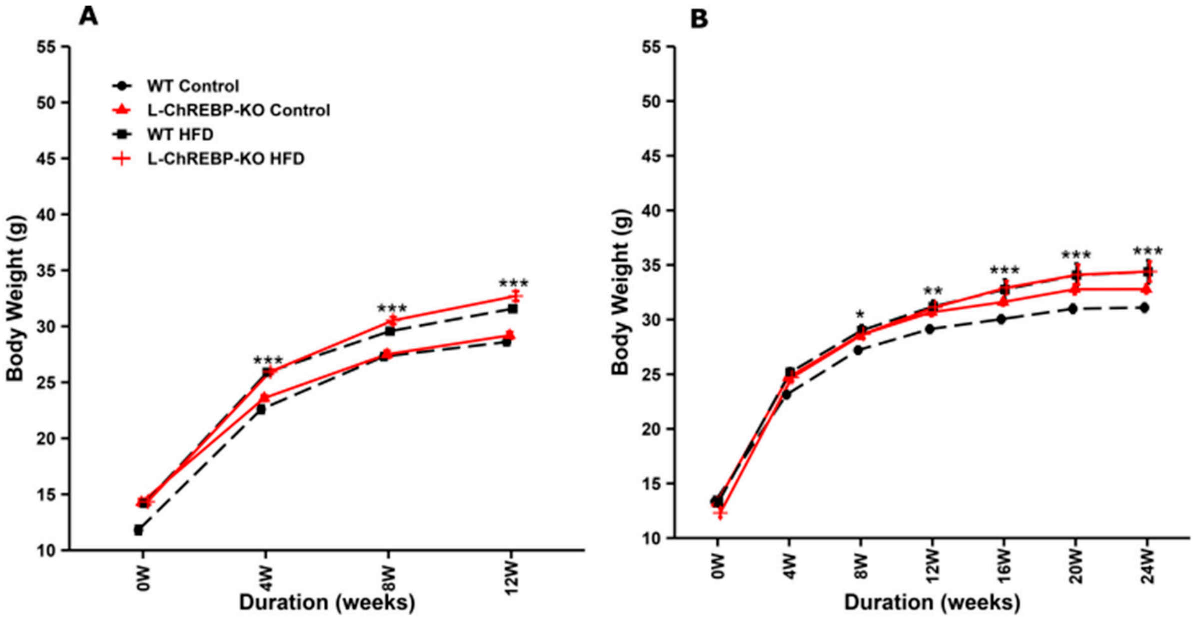

Supplementary Figure S2: Body weight of WT and L-ChREBP-KO mice after 12-week and 24-week. (**A**). Changes in body weight in 12-week period (n = 64 per group in HFD; n = 25 per group in control). (**B**). Body weight in 24 weeks (n = 17 per group in HFD; n = 25 per group in control). Values of the data are expressed as mean ± SEM. Significant differences are indicated as follows: *P < 0.05, **P < 0.01 and ***P < 0.01 vs. the control group.

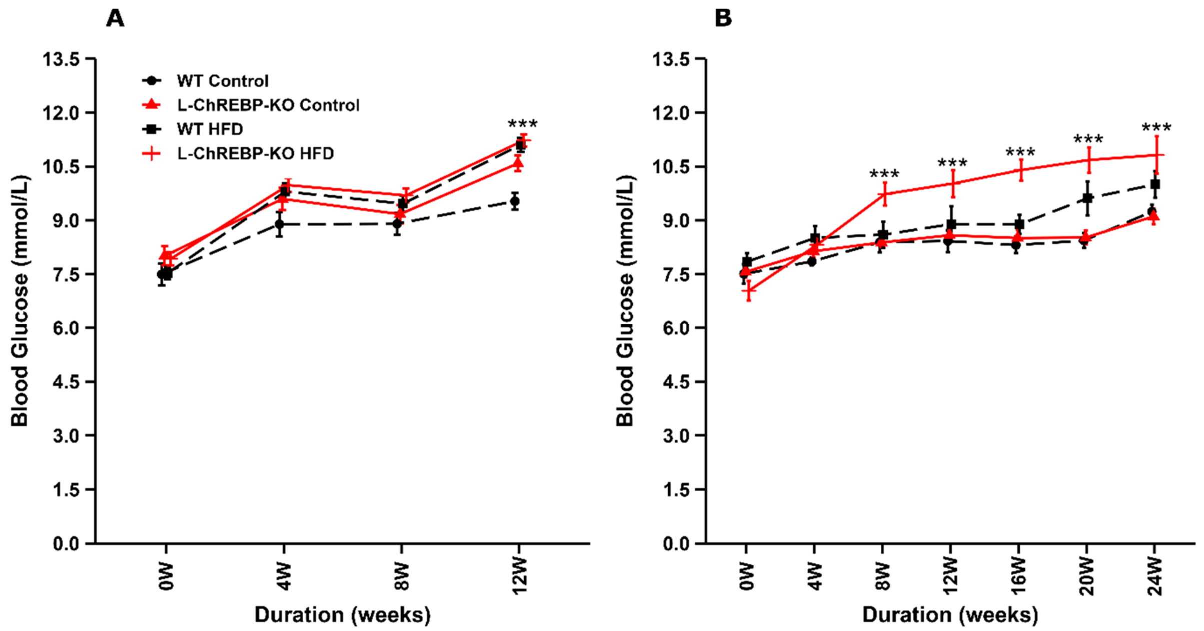

Supplementary Figure S3: Basal blood glucose levels in WT and L-ChREBP-KO mice. (**A**). Blood glucose levels in 12-week mice (n = 64 per group in HFD; n = 25 per group in control). (**B**). Changes in blood glucose levels in 24-week period (n = 17 per group in HFD; n = 25 per group in control). Values of the data are expressed as mean ± SEM. Significant differences are indicated as follows: *P < 0.05, **P < 0.01 and ***P < 0.01 vs. the control group.

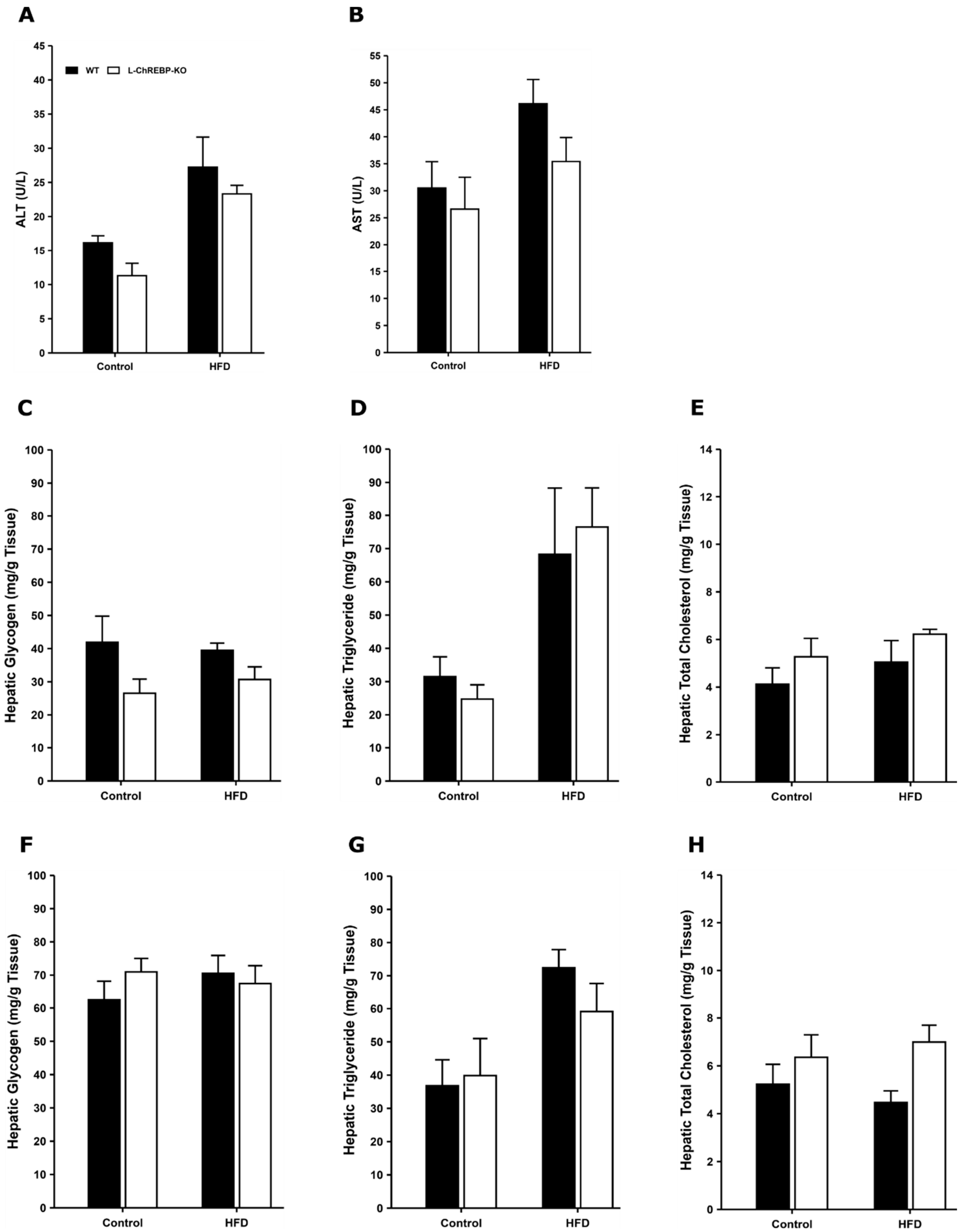

Supplementary Figure S4: Effects of HFD feeding on biochemical parameters in WT and L-ChREBP-KO mice. (A,B) Serum ALT and AST levels in 24-week mice. (**C-E**) Hepatic glycogen, triglyceride and total cholesterol in 12-week period. (**F-H**) Changes in hepatic glycogen, triglyceride and total cholesterol in 24-week mice. n = 4-6 per group. Values of the data are expressed as mean ± SEM. Significant differences are indicated as follows: *P < 0.05, **P < 0.01 and ***P < 0.01 vs. the control group.

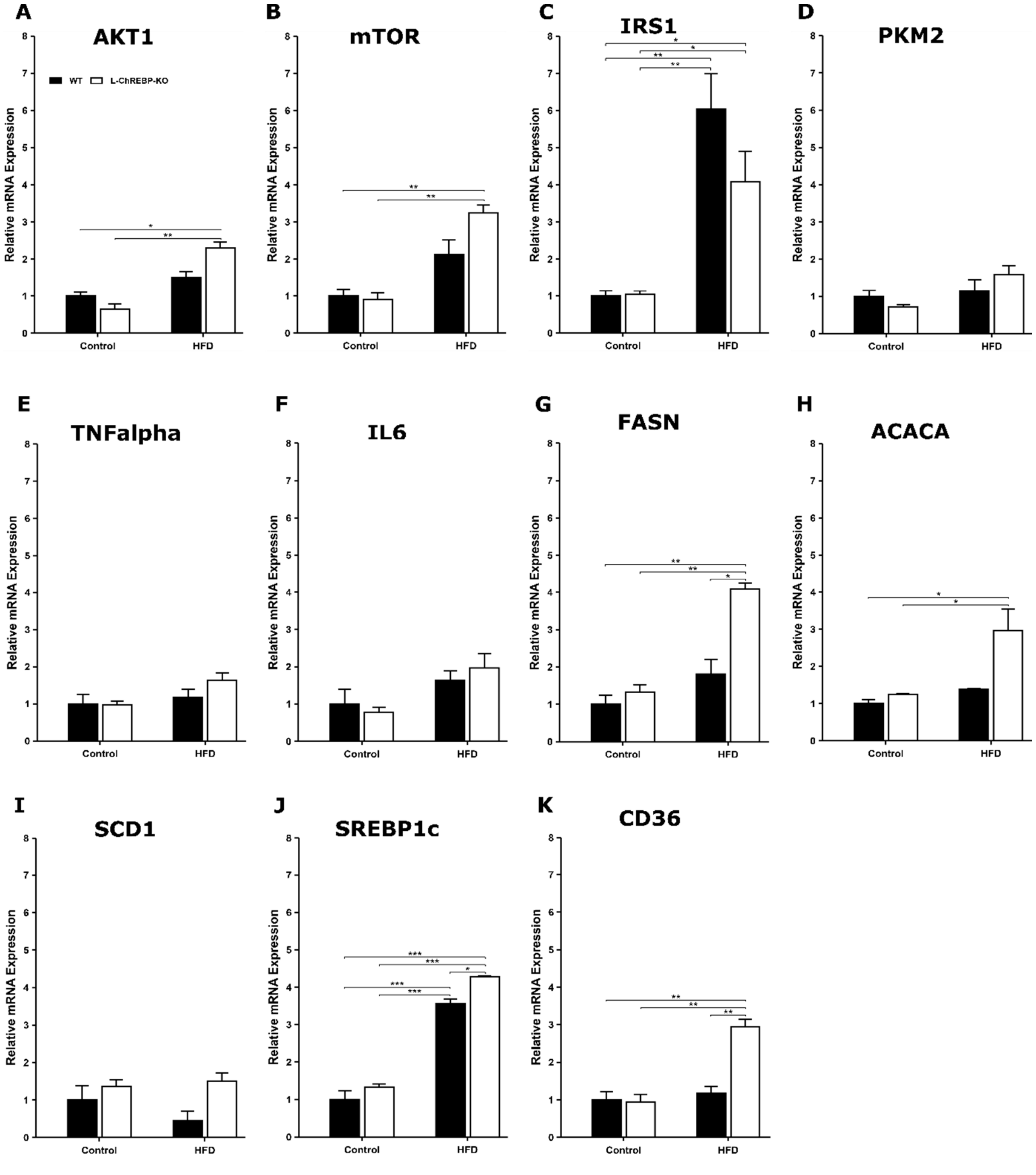

Supplementary Figure S5: Effects of HFD on the gene expression levels of AKT/mTOR pathway, insuling signalling substrate, glycolysis, de novo lipogenesis and fat transportation in 12-week mice. (**A,B**) mRNA levels of AKT1 and mTOR. (**C,D**) Gene expression levels of IRS1 and PKM2. (**E,F**) mRNA levels of inflammatin marker TNFalpha and IL6. (**G-K**) Gene expression levels of lipogenesis candidates (FASN, ACACA, SCD1, SREBP1c) and fat transport CD36 gene. n = 3-4 per group. Values of the data are expressed as mean ± SEM. Significant differences are indicated as follows: *P < 0.05, **P < 0.01. and ***P < 0.01 vs. the control group.

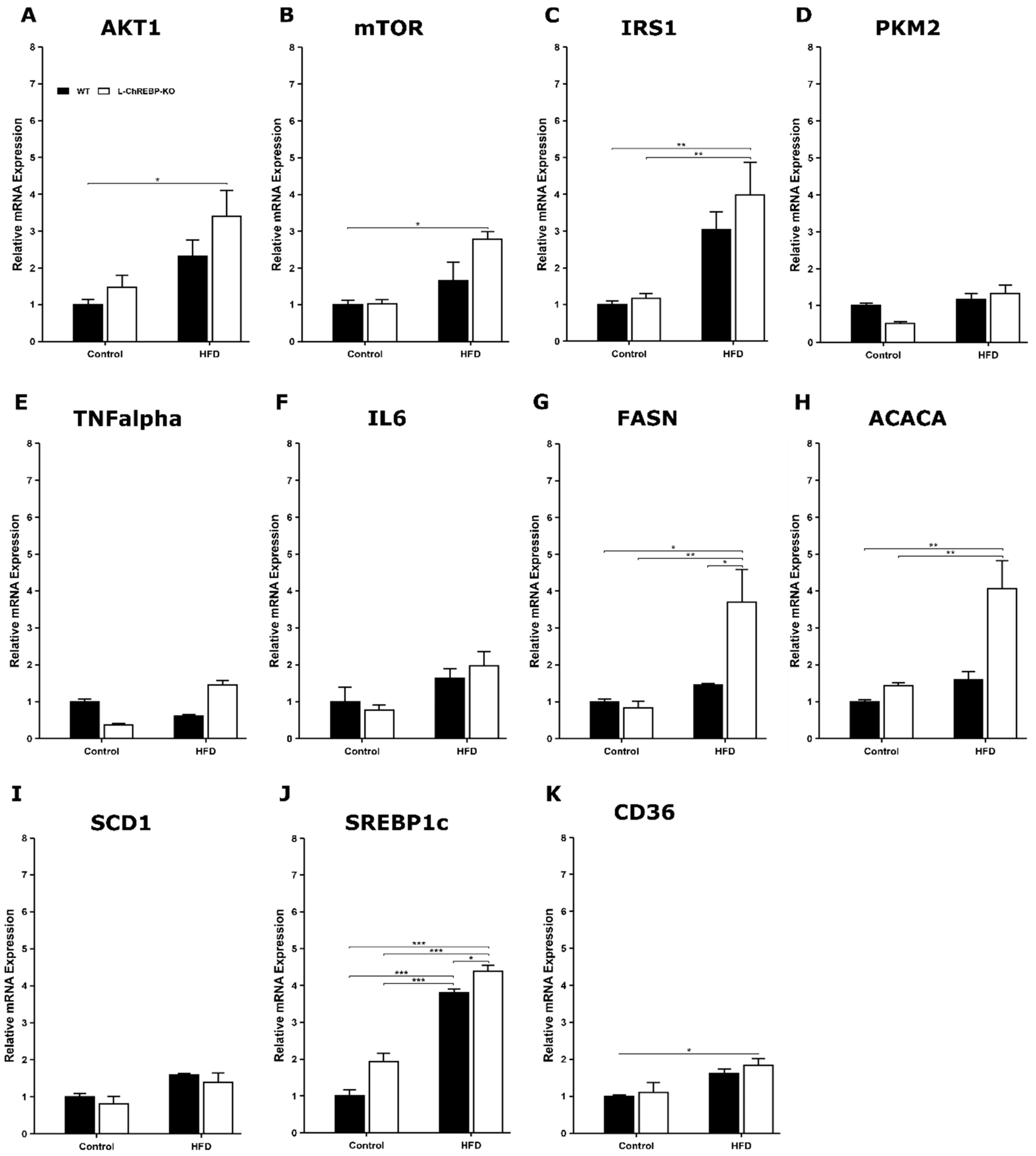

Supplementary Figure S6: Effects of HFD on mRNA levels of AKT/mTOR pathway, insuling signalling substrate, glycolysis, de novo lipogenesis and fat transportation in 24-week mice. (**A,B**) mRNA levels of AKT1 and mTOR (**C,D**) Gene expression levels of IRS1 and PKM2. (**E,F**) mRNA levels of inflammatin marker TNFalpha and IL6. (**G-K**) mRNA levels of lipogenesis candidates (FASN, ACACA, SCD1, SREBP1c) and fat transport CD36 gene. n = 3-4 per group. n = 3-4 per group. Values of the data are expressed as mean ± SEM. Significant differences are indicated as follows: *P < 0.05, **P < 0.01 and ***P < 0.01 vs. the control group.

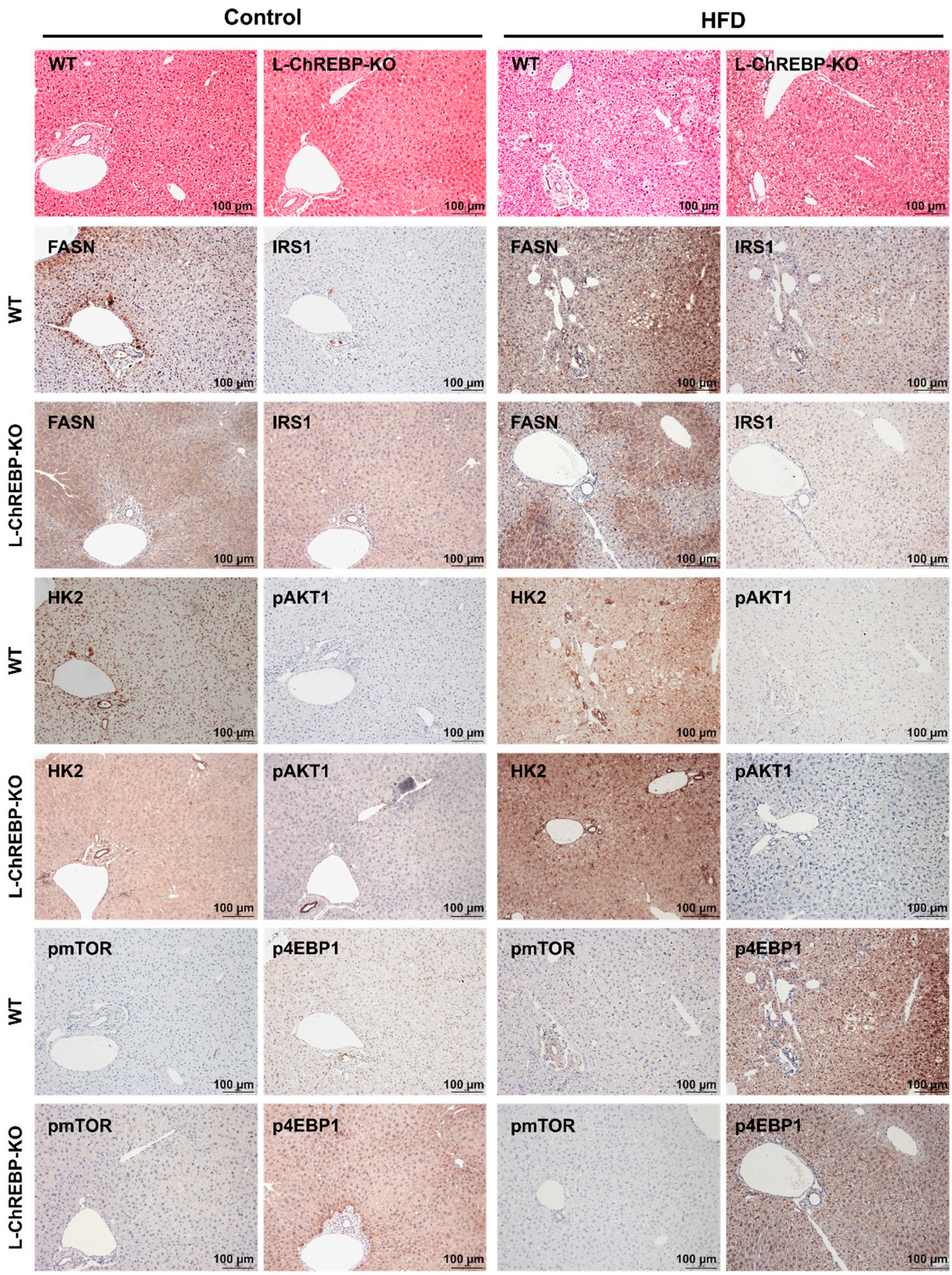

Supplementary Figure S7: Representative images of immunohistochemical staining in liver tissues of WT and L-ChREBP-KO at 12 weeks. Alteration of AKT/mTOR pathway, glycolytic pathway HK2, PKM2 ,and insulin signaling IRS1 and de novo lipogenesis candidates FASN expression. Scale bar: 100 µm.

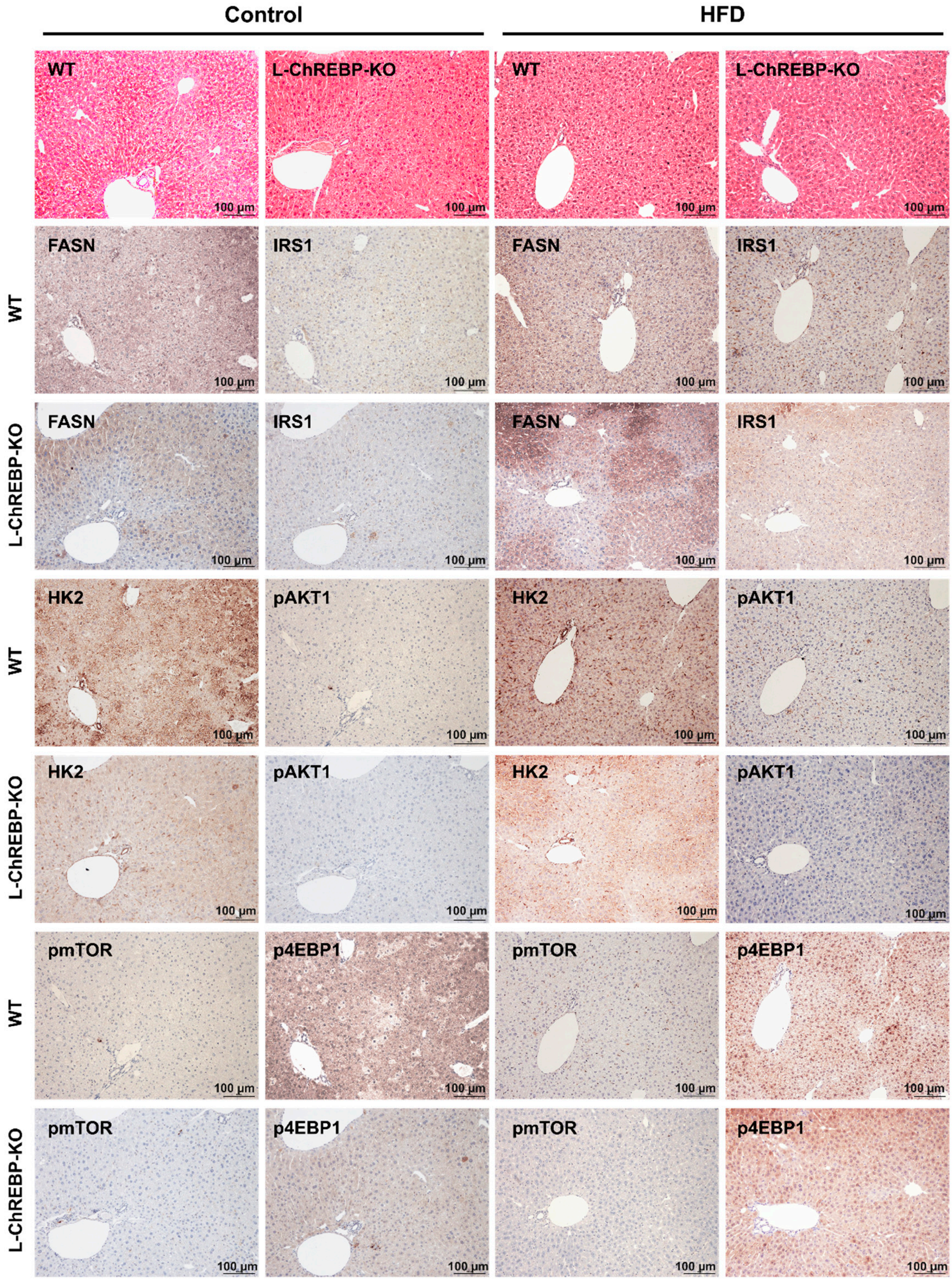

Supplementary Figure S8: Representative images of immunohistochemical staining in liver tissues of WT and L-ChREBP-KO at 24 weeks. Alteration of AKT/mTOR pathway, glycolytic pathway HK2, PKM2 ,and insulin signaling IRS1 and de novo lipogenesis candidates FASN expression. Scale bar: 100 µm.

Supplementary Table S4: Hepatocyte proliferation in normal liver tissue. Proliferative activity as indicated by Ki-67 index in normal liver parenchyma in WT, L-ChREBP-KO and systemic ChREBP-KO mice is shown.

Groups	12 Weeks (%)	24 Weeks (%)	48 Weeks (%)
ChREBP WT Control	0.37 ± 0.05	0.31 ± 0.06	0.21±0.03
L-ChREBP-KO Control	0.24 ± 0.02	0.22 ± 0.03	0.32±0.03
ChREBP WT HFD	0.41 ± 0.05#	0.60 ± 0.09*#	1.03±0.09*#
L-ChREBP-KO HFD	0.55 ± 0.05#	0.35 ± 0.04#	1.19±0.10*#

** P<0.05 vs WT Control; # P<0.05 vs L-ChREBP-KO Control.*

Supplementary Table S5: Tumor incidence in WT and L-ChREBP-KO mice

Genotype	Total (n)	HCC	HCA	HCA Frequency (%)	HCC Frequency (%)	Fisher Exact Test
WT	264	4	11	4.17	1.51	ns
L-ChREBP-KO	264	1	7	2.65	0.37	

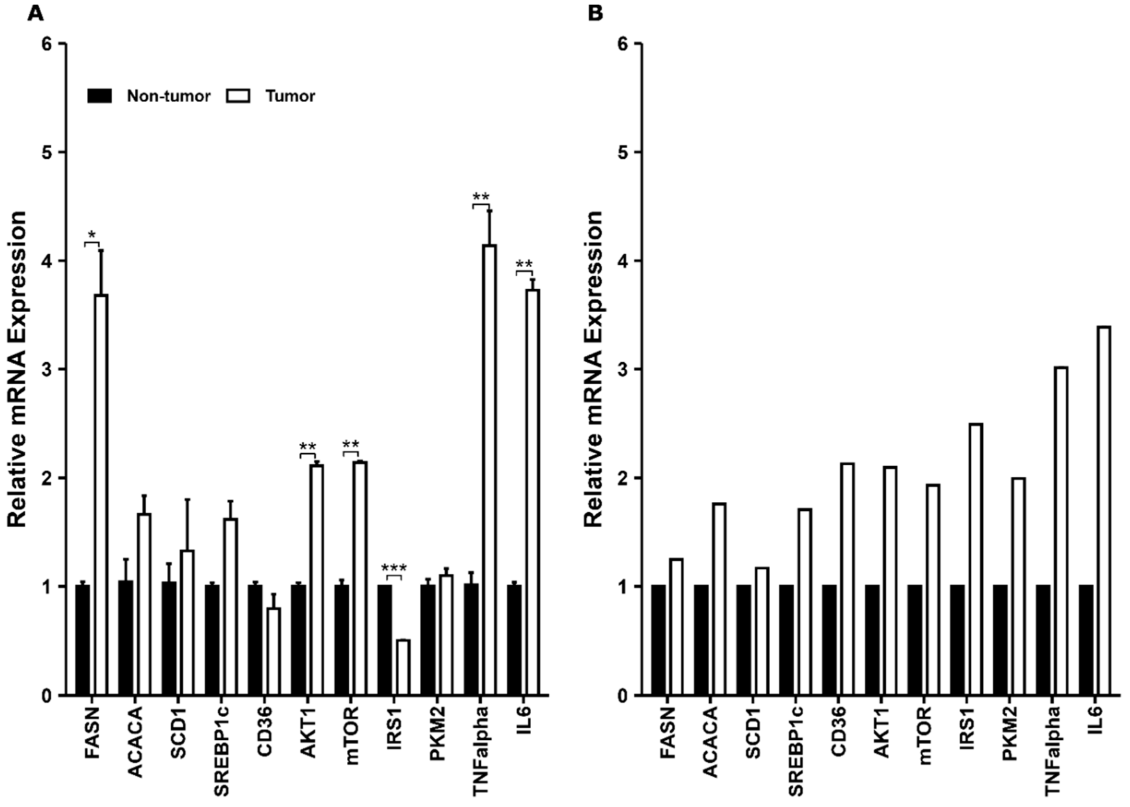

Supplementary Figure S9: Gene expression levels in tumor and non-tumor liver tissue. (**A**). Effects of HFD on WT mice in HCC development. (**B**). Changes in gene expression levels de novo lipogenesis, AKT/mTOR, IRS1, glycolysis, and inflammation marker IL6 and TNFalpha in L-ChREBP-KO mice during HCC development. Statistical test was not performed in L-ChREBP-KO mice becausee of having single sample. Values of the data are expressed as mean ± SEM. Significant differences are indicated as follows: *P < 0.05, **P < 0.01 and ***P < 0.01 vs. the control group.
